# The Zinc Content of HIV-1 NCp7 Affects Its Selectivity for Packaging Signal and Affinity for Stem-Loop 3

**DOI:** 10.3390/v13101922

**Published:** 2021-09-24

**Authors:** Ying Wang, Chao Guo, Xing Wang, Lianmei Xu, Rui Li, Jinzhong Wang

**Affiliations:** 1 TEDA Institute of Biological Sciences and Biotechnology, Nankai University, 23 Hongda Street, TEDA, Tianjin 300457, China; yingwang@nankai.edu.cn (Y.W.); 2120191118@mail.nankai.edu.cn (X.W.); 2120191119@mail.nankai.edu.cn (L.X.); 2120201150@mail.nankai.edu.cn (R.L.); 2 Key Laboratory of Molecular Microbiology and Technology, Ministry of Education, 23 Hongda Street, TEDA, Tianjin 300457, China; 3 Tianjin Key Laboratory of Microbial Functional Genomics, 23 Hongda Street, TEDA, Tianjin 300457, China; 4 College of Basic Medical Sciences, Shanxi Medical University, Taiyuan 030001, China; guochaotju@126.com

**Keywords:** HIV-1, NCp7, zinc, Psi-selectivity, SL3-affinity

## Abstract

The nucleocapsid (NC) protein of human immunodeficiency (HIV) is a small, highly basic protein containing two CCHC zinc-finger motifs, which is cleaved from the NC domain of the Gag polyprotein during virus maturation. We previously reported that recombinant HIV-1 Gag and NCp7 overexpressed in an *E. coli* host contains two and one zinc ions, respectively, and Gag exhibited much higher selectivity for packaging signal (Psi) and affinity for the stem-loop (SL)-3 of Psi than NCp7. In this study, we prepared NCp7 containing 0 (^0^NCp7), 1 (NCp7) or 2 (^2^NCp7) zinc ions, and compared their secondary structure, Psi-selectivity and SL3-affinity. Along with the decrease of the zinc content, less ordered conformations were detected. Compared to NCp7, ^2^NCp7 exhibited a much higher Psi-selectivity and SL3-affinity, similar to Gag, whereas ^0^NCp7 exhibited a lower Psi-selectivity and SL3-affinity, similar to the H23&H44K double mutant of NCp7, indicating that the different RNA-binding property of Gag NC domain and the mature NCp7 may be resulted, at least partially, from their different zinc content. This study will be helpful to elucidate the critical roles that zinc played in the viral life cycle, and benefit further investigations of the functional switch from the NC domain of Gag to the mature NCp7.

## 1. Introduction

The nucleocapsid (NC) protein of human immunodeficiency (HIV), designated as NCp7, is a small highly basic protein, which contains two conserved CCHC zinc-finger motifs and flanked basic residues. NCp7 is synthesized as a domain (NC domain) of the Gag polyprotein [1]. The NC domain of Gag is responsible for the specific recognition and selection of viral packaging signal (Psi, ψ) located at the 5′-leader of the viral genome from a substantial excess of cellular RNAs, resulting in the encapsidation of two copies of genome RNA into the viral particle [2,3,4]. Shortly after virus release from the cell, NCp7 is liberated by viral protease-mediated cleavage of the Gag polyprotein during virus maturation [5,6]. NCp7 is regarded as an ATP-independent nucleic acid (NA) chaperone. About 1500~2000 NCp7 molecules are present per viral core, extensively coating and protecting the entire viral genome RNA in a histone-like manner [7]. It is not clear how the same protein, alone or as part of Gag, performs different RNA binding functions in the viral life cycle [8,9].

Zinc is the second-most abundant trace metal in the human body, and is a component of ~10% of the human proteome [10], playing essential roles in a wide variety of biological processes [11]. Although the significance of zinc status in HIV infection has not been fully elucidated, HIV is regarded as a zinc-dependent virus [12,13]. Zinc is an integral component of HIV Gag and NCp7. Amino acid mutations that prevent Zn^2+^ binding to the zinc-finger motifs of Gag inhibit genome RNA packaging and lead to noninfectious viruses [14,15]. Similarly, compounds that eject zinc from zinc-fingers of Gag inhibit viral infection [16,17]. Besides, zinc is also coordinated by HIV-1 integrase [18], Vif [19], Tat [20] and HIV-2 Vpx [21], and inhibits HIV-1 protease [22] and reverse transcriptase (RT) [23].

We previously reported that recombinant HIV-1 Gag and NCp7 overexpressed in an *E. coli* host contains two and one zinc ions respectively, and Gag exhibited much higher selectivity for Psi-RNA and affinity for the stem-loop (SL) 3 of Psi than NCp7 [24]. In this study, NCp7 with or without two zinc ions were generated by incubating NCp7 at 100 °C and subsequently cooling down rapidly (^0^NCp7), or slowly in the presence of excess Zn^2+^ (^2^NCp7). ^0^NCp7, NCp7 and ^2^NCp7 share the same amino acid sequence, but with different zinc content. We compared the secondary structure, Psi-selectivity and SL3-affinity of ^0^NCp7, NCp7 and ^2^NCp7, and found that along with the decrease of zinc content, less ordered secondary structure, and much lower Psi-selectivity and SL3-affinity were detected. These results confirmed that zinc content affects the RNA selection and binding of NCp7, indicating that the different zinc coordination of Gag NC domain and mature NCp7 may be the reason, at least partially, for their different RNA-binding property and function.

## 2. Materials and Methods

### 2.1. Protein Purification and Heat-Treatment

*E. coli* BL21 (DE3) was transformed with pET28-NCp7, and 6× histidine tagged NCp7 was purified by nickel affinity chromatography as described [25]. Protein concentration was determined by bicinchoninic acid assay (Thermo Scientific Pierce, Rockford, IL, USA). Purified NCp7 was incubated at 100 °C for 0, 5, 10, 30 or 60 min, respectively, then cooled down rapidly using an ice bath or slowly at 1 °C/min using a T-Gradient thermocycler (Biometra, Göttingen, Germany). Otherwise, purified NCp7 was supplemented with excess zinc sulfate (10-fold molar concentration of NCp7), incubated at 100 °C for 0, 5, 10, 30 or 60 min, respectively, and cooled down slowly at 1 °C/min to 25 °C. Samples were then centrifuged to remove precipitated proteins and dialyzed overnight.

### 2.2. Determination of Zn^2+^ Content

Each of the heat-treated NCp7 samples were dialyzed overnight against 1×HEPES and diluted to 25 μM. The Zn^2+^ content of each protein was analyzed via a spectrophotometric method using the 4-(2-pyridylazo) resorcinol (PAR) assay according to the protocol described by Doyle et al. [26]. The Zn^2+^ content of ^0^NCp7, NCp7 and ^2^NCp7 were also confirmed using inductively coupled plasma-optical emission spectrometer (ICP-OES) on the Spectro analytical instruments (Kleve, Germany). The emission line of scanned Zn^2+^ was 213.856 nm [27].

### 2.3. Cell Culture, Transfection and RNA Preparation

293T cells were cultured in Dulbecco’s modified Eagle medium (HyClone, Logan, UT, USA) supplemented with 10% fetal bovine serum and 1% penicillin-streptomycin at 37 °C with 5% CO_2_. Cells were transfected with 3 µg pCMV-Tag2-Psi using the polyethylenimine reagent (Polysciences, Warrington, PA, USA), and were harvested at 72 h post-transfection. Total RNA was isolated using TRIzon Regent (CoWin Biosciences, Beijing, China) as described [24]. RNase-free DNase I (TaKaRa, Otsu Shiga, Japan) was utilized to eliminate DNA contamination. RNA was dissolved in RNase-free water and quantified on a NanoDrop^®^ ND-2000 Spectrophotometer (ThermoFisher, Waltham, MA, USA).

### 2.4. In Vitro Protein-RNA Binding and RT-qPCR

A total of 5 µg RNA was incubated with 15 µg purified protein for 2–3 min on ice in binding buffer (10 mM HEPES, pH 7.9, 50 mM potassium chloride, 0.5 mM dithiothreitol, 1 mM magnesium chloride and 0.5 mM phenylmethanesulfonyl fluoride). RNase A (20 µg/mL) was utilized to remove unbound RNA at 37 °C for 10 min. RNA in the protein-RNA complex was subsequently extracted and quantified by RT-qPCR. First-strand cDNA was synthesized using HIV-1 reverse transcriptase p66/p51, and qPCR was conducted using UltraSYBR Mastermix (CoWin Biosciences) and ABI 7500 Fast Real-Time PCR system (Applied Biosystems, Foster City, CA, USA) as described [25]. The threshold cycle (*C_T_*) values were collected from at least three biological replicates, and the relative amount of Psi-RNA was calculated using the cycle threshold method (2^−∆∆*CT*^) and β-actin RNA as a reference for normalization [28].

### 2.5. The Far-UV Circular Dichroism (CD) and Fluorescence Spectroscopic Analyses

Proteins were dialyzed overnight against 1× PBS and adjusted to 0.2 μg/μL. CD measurements were performed with a Jasco J-715 spectropolarimeter (Jasco, Tokyo, Japan) over the wavelength range of 190–260 nm. A 1 mm path length quartz cuvette was utilized and all spectra were recorded with a scan rate of 100 nm/min, a bandwidth of 1 nm, and a detector response time of 2 s. Fluorescence measurements were performed with a Hitachi F-7000 fluorescence spectrophotometer (Hitachi, Tokyo, Japan). Protein samples were adjusted to 0.1 μg/μL and were transferred into a 1 cm path length cuvette. The excitation wavelength was set at 280 nm by scanning the emission spectra between 300 and 450 nm. The slit width for excitation and emission spectra was 5 nm.

### 2.6. Surface Plasmon Resonance (SPR) Experiments on Biacore

SPR measurements were performed using streptavidin-coated sensor chips (SA chip) on a Biacore^TM^ X100 analytical system (GE Healthcare, Uppsala, Sweden). Around 50 resonance units (RU) of 3′-biotinylated SL3-RNA probe (5′-GGACUAGCGGAGGCUAGUCC-3′) [29] were immobilized on the SA chip surface. Proteins were dialyzed overnight against HBS-T buffer (10 mM HEPES, pH 7.4, 150 mM sodium chloride and 0.005% Tween-20), and five different concentrations of protein were prepared by serial dilution in HBS-T buffer for each set of sensorgrams. The samples were injected at a flow rate of 30 µL/min. The injection step included a 60 s association phase followed by a 180 s dissociation phase in HBS-T buffer. All binding experiments were performed at 25 °C. At the end of each cycle, the chip surface was regenerated by the injection of 0.1% SDS for the complete dissociation of proteins from the RNA probe. Response unit values were recorded at intervals of 1 s. The sensorgrams were processed for baseline alignment and reference channel subtraction with the Biacore^TM^ X100 Evaluation Software (GE Healthcare). Kinetic analysis was performed by globally fitting the curves describing a simple 1:1 bimolecular model to the set of five sensorgrams [30]. The quality of data set and reliability of the global fittings were verified automatically by the software. The closeness of fit is judged by chi-square value, which describes the deviation between the experimental and fitted curves. The significance of the kinetic parameter values returned by the fitting procedure is indicated by standard deviations generated from two independent experiments.

## 3. Results

### 3.1. Generation of NCp7 without or with Two Zinc Ions

We previously reported that recombinant HIV-1 Gag and NCp7 overexpressed in *E. coli* contains two and one zinc ions, respectively [24]. We also found that NCp7 is thermostable, and the nucleic acid (NA)-binding activity of NCp7 at high NC:NA ratios is independent on its zinc-fingers [25]. Therefore, in this study, we detected the Zn^2+^ content of thermo-stressed NCp7 (Table 1). The purified NCp7 was incubated at 100 °C for 5, 10, 30 or 60 min, respectively. If the thermo-stressed NCp7 were cooled down rapidly, the Zn^2+^-to-protein ratios are 0.49 (5 min), 0.34 (10 min), 0.01 (30 min) and 0.01 (60 min), respectively, demonstrating that the heat-denatured NCp7 can hardly be refolded under this condition. However, if the thermo-stressed NCp7 was cooled down slowly at 1 °C/min, the Zn^2+^-to-protein ratios are 0.97 (5 min), 0.96 (10 min), 0.97 (30 min) and 0.94 (60 min), respectively, similar to that of NCp7 without thermo-stress (0.97). If the NCp7 was cooled down slowly in the presence of excess zinc sulfate after being incubated at 100 °C, the Zn^2+^-to-protein ratio increased to 1.68 (5 min), 1.87 (10 min), 1.98 (30 min) and 1.85 (60 min), respectively, indicating that both zinc-finger motifs were metalated with Zn^2+^ when the heat-denatured NCp7 was refolded under this condition. By doing so, we were able to generate NCp7 with or without two zinc ions. The Zn^2+^ contents of NCp7 incubated at 100 °C for 30 min and cooled down rapidly, or slowly in excess Zn^2+^ were confirmed by inductively coupled plasma-optical emission spectrometer (ICP-OES). Their Zn^2+^-to-protein ratios are 0.004 ± 0.005 and 1.99 ± 0.13, respectively, and they were designated as ^0^NCp7 and ^2^NCp7. High temperature denaturation did not change the conformation of NCp7 permanently, since both CD [25] and fluorescence spectra (Figure S1) recovered after NCp7 was incubated at 100 °C for 5, 10, 30 or 60 min, respectively, and then cooled down slowly to 25 °C. Therefore, the NCp7 without thermo-stress was utilized in further investigations as a control; its Zn^2+^-to-protein ratio was also confirmed by ICP-OES to be 0.97 ± 0.08.

### 3.2. Comparison of the Conformation of ^0^NCp7, NCp7 and ^2^NCp7

**Figure 1:**
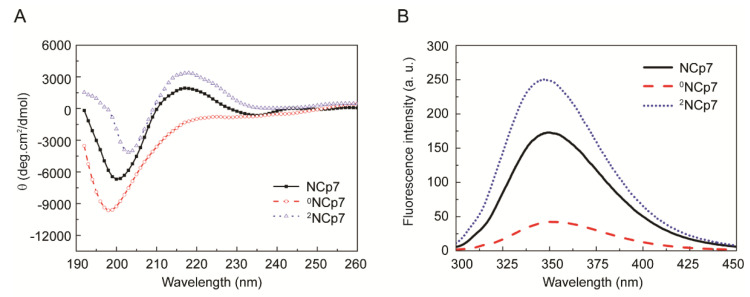
Comparison of ^0^NCp7, NCp7 and ^2^NCp7 in the secondary structure. (**A**) Circular dichroism spectra of ^0^NCp7, NCp7 and ^2^NCp7. (**B**) Fluorescence spectra of ^0^NCp7, NCp7 and ^2^NCp7.

We next sought to compare the conformation of ^0^NCp7, NCp7 and ^2^NCp7 by CD and fluorescence spectroscopic analyses (Figure 1). The CD spectrum of NCp7 exhibits a broad band with positive maximum centered at ~216 nm and a strong band with negative minimum centered at ~200 nm, consistent with those in literatures [31,32]. The removal of Zn^2+^ from NCp7 disrupted the protein structure, switching the signal at ~216 nm from positive to negative. Moreover, a significant increase in the intensity of the negative peak as well as a slight blue shift of the minimum ellipticity were detected (Figure 1A, ^0^NCp7), indicative of a less ordered structure [33]. This spectrum is similar to those of EDTA-treated and H23&44K double mutant of NCp7 we previously reported [25]. When the ^2^NCp7 was tested, an increase of the positive ellipticity and a decrease of the negative ellipticity, as well as a slight red shift of the minimum ellipticity were detected (Figure 1A, ^2^NCp7). The increase of ordered structure along with the increase of Zn^2+^ content suggested that zinc coordination may be crucial for the polypeptide chain folding and maintaining the ordered structure of NCp7.

NCp7 contains a tryptophan residue (Trp37) and exhibits intrinsic fluorescence with excitation at 280 nm. The fluorescence spectra of ^0^NCp7, NCp7 and ^2^NCp7 were detected (Figure 1B). Compared to that of NCp7, the fluorescence intensity of ^2^NCp7 increased by 43.8% whereas that of ^0^NCp7 decreased by 75.6%. Since fluorescence is critically dependent on the atomic environment of tryptophan and on its accessibility to solvent, and changes in fluorescence frequently acts as a sensitive monitor of widespread conformational change [34], these results, together with those of CD, can be interpreted to indicate that Zn^2+^ stabilized the ordered structure of NCp7.

### 3.3. Comparison of ^0^NCp7, NCp7 and ^2^NCp7 in Their Selectivity for Psi-RNA

**Figure 2:**
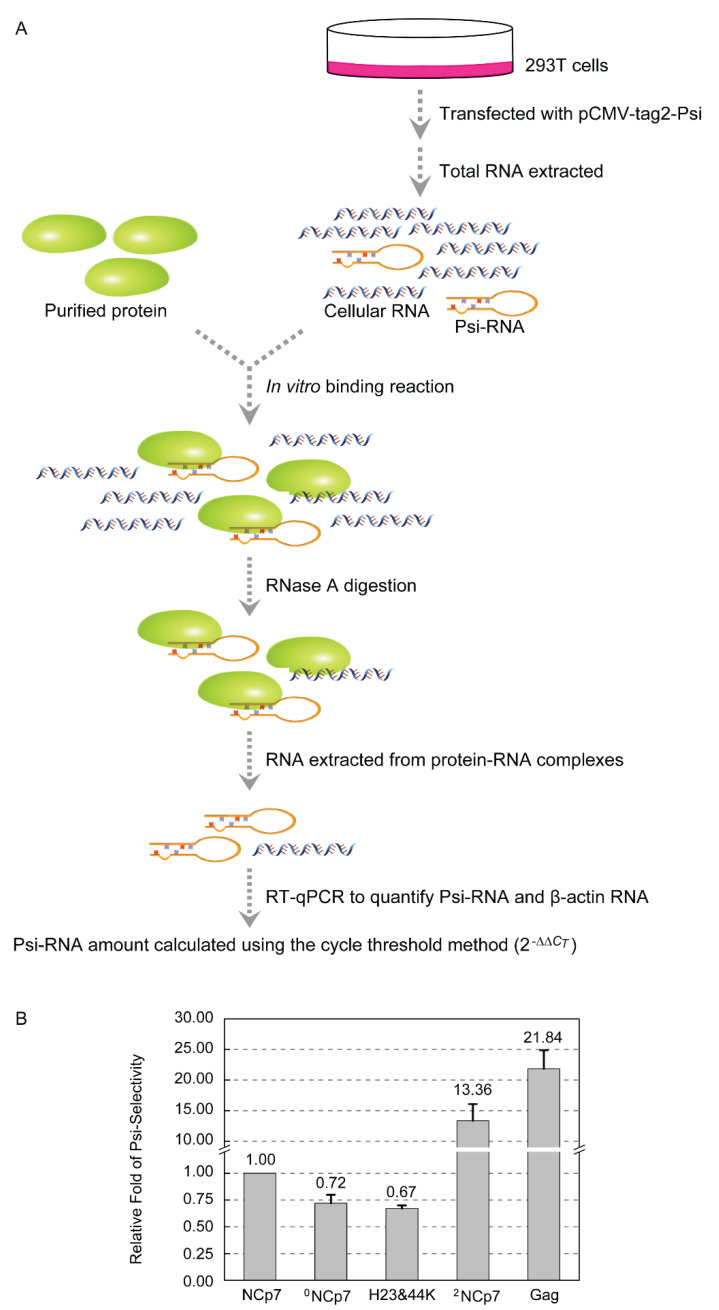
Comparison of ^0^NCp7, NCp7 and ^2^NCp7 in the Psi-selectivity. (**A**) Schematic representation of the approach to quantifying the Psi-RNA selected by ^0^NCp7, NCp7 and ^2^NCp7 from in vitro binding reactions. (**B**) The Psi-selectivity of ^0^NCp7, NCp7 and ^2^NCp7. The data are means and standard deviations from three independent experiments.

We next asked whether zinc content of NCp7 affects its RNA-binding property. We previously established an RT-qPCR-based strategy to quantitatively compare the selectivity of Gag and NCp7 for the HIV-1 Psi in *E. coli* [24]. Here, the selectivity of ^0^NCp7, NCp7 and ^2^NCp7 for Psi-RNA was compared using a similar approach (Figure 2A). 293T cells were transfected with pCMV-tag2-Psi, and total RNA was isolated and allowed to incubate with ^0^NCp7, NCp7 and ^2^NCp7, respectively, in the in vitro binding reactions. Unbound RNA was removed and the Psi-RNA that each protein associated with in the protein-RNA complexes was quantified by RT-qPCR, using β-actin RNA that the protein bound as a reference for normalization. If the normalized amount of Psi-RNA that NCp7 selected in the presence of 293T total RNA was designated as 1, the selectivity of ^0^NCp7 for Psi-RNA was 0.72, similar to that of the H23&44K double mutant of NCp7 (0.67). However, the selectivity of ^2^NCp7 and Gag for Psi-RNA were 13.36 and 21.84, respectively, much higher than those of NCp7 and ^0^NCp7 (Figure 2B). These results confirmed our previous finding that Gag exhibited a much higher Psi-selectively than NCp7, and demonstrated that Zn^2+^ coordination on the zinc-finger motif is crucial for the selection of Psi-RNA among a large variety of cellular RNAs. Zinc content did not affect the nonspecific RNA-binding ability of NCp7, because the *C_T_* values were 26.09, 26.21 and 26.12, respectively, when the β-actin RNA that ^0^NCp7, NCp7 or ^2^NCp7 associated with in the protein-RNA complexes were amplified by RT-qPCR.

### 3.4. Comparison of ^0^NCp7, NCp7 and ^2^NCp7 in Their Binding Affinity to SL3-RNA

**Figure 3:**
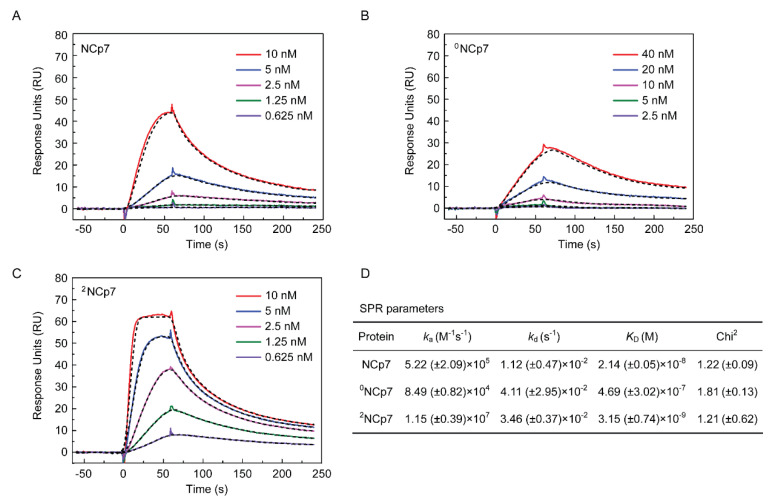
Kinetic analyses of ^0^NCp7, NCp7 and ^2^NCp7 binding to SL3-RNA, determined by SPR. Representative SPR kinetic curves and fits for (**A**) NCp7, (**B**) ^0^NCp7 and (**C**) ^2^NCp7 binding to SL3-RNA. The amount of SL3-RNA immobilized on the chip surface was 50 RU (A, C) and 100 RU (B), respectively. The concentrations of NCp7 and ^2^NCp7 are 0.625, 1.25, 2.5, 5 and 10 nM, whereas the concentrations of ^0^NCp7 are 2.5, 5, 10, 20 and 40 nM. Experimental and fitted curves are colored and black, respectively. (**D**) SPR parameters of ^0^NCp7, NCp7 and ^2^NCp7 binding to SL3-RNA, generated from two independent experiments, with standard deviations in brackets. Chi-square values are also shown, which describe the deviation between the experimental and fitted curves.

The binding affinity of ^0^NCp7, NCp7 and ^2^NCp7 to the SL3 of Psi was analyzed using the SPR technique (Figure 3). Around 50 RU of biotinylated SL3-RNA probe was immobilized on the SA chip, and the SPR responses reached to about 45 and 65 RU for NCp7 (Figure 3A) and ^2^NCp7 (Figure 3C), respectively, when the protein concentrations were increased to 10 nM. However, ^0^NCp7 showed a much lower extent of SPR response at the same concentration. Such low SPR response challenged to accurately quantify the interaction of ^0^NCp7 with SL3-RNA by fitting curves. Therefore, 100 RU immobilized SL3-RNA probe and increased protein concentrations were utilized for ^0^NCp7, and the SPR response reached to about 30 RU at the protein concentration of 40 nM (Figure 3B), indicating that the binding affinity of ^0^NCp7 is much lower than those of NCp7 and ^2^NCp7. More rapid binding of ^2^NCp7 to SL3-RNA was detected than NCp7, with an association rate constant (*k*_a_) of 1.15 × 10^7^ M^−1^s^−1^, a dissociation constant (*k*_d_) of 3.46 × 10^−2^ s^−1^, and a resulting dissociation equilibrium constant (*K*_D_) of 3.15 × 10^−9^ M. For NCp7 and ^0^NCp7, the *k*_a_, *k*_d_, and *K*_D_ are 5.22 × 10^5^ and 8.49 × 10^4^ M^−1^s^−1^, 1.12 × 10^−2^ and 4.11 × 10^−^^2^ s^−1^, 2.14 × 10^−8^ and 4.69 × 10^−7^ M, respectively (Figure 3D). The correlation between experimental and fitted sensorgrams was observed and the chi-square values were all below two, indicating a reliable closeness of fit between the experimental and fitted curves. These results showed that the binding affinity of ^2^NCp7 to SL3-RNA is about one order of magnitude higher than NCp7, and that of NCp7 is about one order of magnitude higher than ^0^NCp7, indicating that Zn^2+^ coordination on the zinc-finger motif is crucial for the high-affinity binding to SL3. Notably, the *K*_D_ value of ^2^NCp7 binding to SL3 is similar to that of Gag (1.75 × 10^−9^ M) and the *K*_D_ value of ^0^NCp7 is similar to that of H23&44K (7.31 × 10^−7^ M) [24].

## 4. Discussion

We previously reported that recombinant Gag and NCp7 overexpressed in *E. coli* contains two and one zinc ions respectively, and the coordination of both zinc ions is essential for the Psi-binding and selection of Gag [24]. In this study, ^0^NCp7, NCp7 and ^2^NCp7 were generated. They contain the same amino acid sequence, but with different zinc content. We showed that, along with the decrease of zinc content, less ordered secondary structures were detected, and the selectivity for Psi-RNA and the binding affinity to SL3-RNA decreased significantly. Among them, ^2^NCp7 is similar to the NC domain of Gag, which contains two Zn^2+^ and can form two functional zinc-fingers necessary for high-affinity binding to SL3 and selection of Psi. However, ^0^NCp7 is similar to the H23&44K double mutant of NCp7 in CD spectrum, Psi-RNA selectivity and SL3-RNA affinity, probably due to their inability to form zinc-fingers. On the other hand, zinc content did not affect the nonspecific binding ability of NCp7 to the β-actin RNA, consistent with our previous findings using EDTA-treated or H23&44K double mutant of NCp7 showing that the NA-binding activity of NCp7 at the NC:NA ratio from 1:1 to 1:10 nt is independent on its zinc-fingers [25]. These results indicated that the different zinc content of Gag NC domain and NCp7 may contribute, at least partially, to their distinct RNA binding property and function, namely, NC domain is responsible for the specific recognition and selection of Psi in the presence of a large variety of cellular RNAs, whereas NCp7, in the mature viral particle, binds genome RNA nonspecifically in a histone-like manner. If this is the case, the removal of one Zn^2+^ during or after the proteolytic cleavages of Gag might be involved in switching the function from NC domain to mature NCp7.

The zinc content of purified HIV-1 has been measured to be about 1.7 mol of Zn^2+^ per mol Gag protein, after the viral preparations were extensively exposed to buffer containing EDTA [35]. It is highly likely that these zinc ions are carried into the virus particle by the two zinc-fingers in the NC domain of Gag at the time of virus assembly, although zinc-binding properties of other viral proteins including integrase [18], Vif [19] and Tat [20] were also reported. After virus maturation, whether all these zinc ions are attached to the NCp7 and whether there exist free zinc ions in the mature viral particle are still unknown.

Both the NC domain of Gag and the mature NCp7 contain two zinc-finger motifs, and are often schematically depicted with two humps. However, whether both zinc-finger motifs, especially in NCp7, bind zinc ions and form functional zinc-finger structures are still unclear. The three-dimensional structure of full-length Gag has not yet been resolved due to its high molecular weight and low solubility, and the longest fragment analyzed by NMR of Gag lacking p2 and p6 [36]. NCp7 is very small, its structure was resolved by NMR and the position of zinc ions was inferred. However, these samples were often obtained by adding zinc ions to the purified NC peptide, either solid-phase synthesized or overexpressed in *E. coli* [37,38,39].

Whether one Zn^2+^ is ejected during or after Gag is proteolytic cleaved to form NCp7 and other structural proteins, and how, awaits further investigation. However, the two zinc-finger motifs of NCp7 did not bind to Zn^2+^ at equal affinity. We previously reported that the zinc contents of H23K and H44K of NCp7 are 0.85 and 0.21, respectively [24], indicating that the N-terminal zinc is more likely to be ejected. Indeed, the two zinc-finger motifs in NCp7 are not equivalent in conformational distribution and accessibility for RNA binding [40]. Small molecules able to eject zinc ions preferentially target the C-terminal zinc-finger rather than the N-terminal one [41]. Currently, we do not know how the NCp7 we used in this study is metalated with one zinc ion. In future investigations, H23K and H44K of NCp7, containing saturated zinc ions, can be utilized.

There are about 1500~2000 Gag molecules present in a premature viral particle. During viral maturation, if a Zn^2+^ is released from each of the Gag molecule, there will be 1500~2000 free zinc ions in the mature virus particle. These free zinc ions may play important roles in the regulation of other viral proteins. For instance, zinc inhibition of HIV-1 protease has been reported, in which Zn^2+^ binds to or in the vicinity of the carboxyl groups of the two catalytic aspartates [22,42]. If there are zinc ions released along with the proteolysis of Gag by viral protease to generate mature NCp7, the activity of protease can be inhibited by the released Zn^2+^, which may serve as a possible negative feedback and finally inactivate the protease after Gag cleavage is completed. Zinc inhibitions of HIV reverse transcriptase (RT) has also been reported [23]. HIV-RT has higher affinity for Zn^2+^ than for Mg^2+^, its integral cofactor. Low concentration of Zn^2+^ can inhibit RT extension in the presence of much higher concentration of Mg^2+^, not by directly stopping catalysis, but by forming a highly stable “dead-end complex” that has profoundly diminished catalytic activity. Notably, the concentration of Zn^2+^ that can inhibit RT is about 2~3 orders of magnitude greater than the level of free available Zn^2+^ in the virus-producing cell [23]. If there are zinc ions released, they may serve as the zinc source to inhibit RT in the virus particle. 

The NCp7 sample we used in this study is overexpressed and purified from *E. coli*, which may be different from the NCp7 produced natively during viral replication by cleaving the Gag polyprotein. Currently, the zinc content of NCp7 which is cleaved from Gag polyprotein is under investigation. Besides, ^0^NCp7 and ^2^NCp7 were generated in this study by controlling the reincorporation of zinc depending on cooling conditions after heat-treatment, and heat-denaturation of NCp7 may have other effects beyond modulating Zn content, although we have reported that high temperature denaturation did not change the conformation of NCp7 permanently [25].

## 5. Conclusions

In this study, ^0^NCp7, NCp7 and ^2^NCp7, containing zero, one or two zinc ions, were generated, and their secondary structure, Psi-selectivity and SL3-affinity were compared. We found that along with the decrease of the zinc content, less ordered secondary structure and much lower Psi-selectivity and SL3-affinity were detected. These results confirmed that zinc content affects the RNA selection and binding of NCp7, indicating that the different zinc coordination of Gag NC domain and NCp7 may be the reason, at least partially, for their different RNA-binding property and function. Our study will be helpful to elucidate the critical roles that zinc played in HIV replication, and benefit further investigations of the functional switch from Gag NC domain to NCp7.

## Figures and Tables

**Table 1:** Zn^2+^-to-protein ratio of treated NCp7.

Time at 100 °C (min)	Cool Down	Zn^2+^-to-Protein Ratio
0	Rapidly	0.98 ± 0.01
5	Rapidly	0.49 ± 0.03
10	Rapidly	0.34 ± 0.02
30	Rapidly	0.01 ± 0.02 ^1^
60	Rapidly	0.01 ± 0.04
		
0	Slowly	0.97 ± 0.01 ^2^
5	Slowly	0.97 ± 0.01
10	Slowly	0.96 ± 0.03
30	Slowly	0.97 ± 0.01
60	Slowly	0.94 ± 0.03
		
0	Slowly in excess Zn^2+^	0.98 ± 0.01
5	Slowly in excess Zn^2+^	1.68 ± 0.04
10	Slowly in excess Zn^2+^	1.87 ± 0.03
30	Slowly in excess Zn^2+^	1.98 ± 0.04 ^3^
60	Slowly in excess Zn^2+^	1.85 ± 0.05

^1^ The Zn^2+^-to-protein ratio of this sample determined by ICP-OES is 0.004 ± 0.005. ^2^ The Zn^2+^-to-protein ratio of this sample determined by ICP-OES is 0.97 ± 0.08. ^3^ The Zn^2+^-to-protein ratio of this sample determined by ICP-OES is 1.99 ± 0.13.

## Supplementary Materials

The following are available online at https://www.mdpi.com/article/10.3390/v13101922/s1, Figure S1: Fluorescence spectra of NCp7 which was incubated at 100 °C for 0, 5, 10, 30 or 60 min, respectively, and cooled down slowly to 25 °C.
