# Supplementary material for: The Zinc Content of HIV-1 NCp7 Affects Its Selectivity for Packaging Signal and Affinity for Stem-Loop 3
Source: Viruses. 2021 Sep 24;13(10):1922. doi: 10.3390/v13101922 (PMC8540335; doi:10.3390/v13101922)
Supplement: Supplementary file 1 [file viruses-13-01922-s001.zip › viruses-1351942-supplementary.pdf]

The Zinc Content of HIV-1 NCp7 Affects Its Selectivity for Packaging Signal and Affinity for Stem-Loop 3

Ying Wang, Chao Guo, Xing Wang, Lianmei Xu, Rui Li and Jinzhong Wang

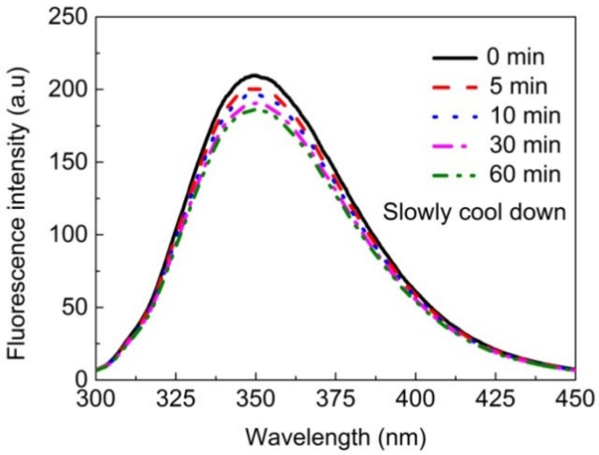

Supplementary Figure S1. Fluorescence spectra of NCp7 which was incubated at 100°C for 0, 5, 10, 30 or 60 min, respectively, and cooled down slowly to 25°C.
